# Composite Hydrogel Model of Cartilage Predicts Its Load-Bearing Ability

**DOI:** 10.1038/s41598-020-64917-1

**Published:** 2020-05-15

**Authors:** Ferenc Horkay, Peter J. Basser

**Affiliations:** Section on Quantitative Imaging and Tissue Sciences, Eunice Kennedy Shriver National Institute of Child Health and Human Development, National Institutes of Health, 13 South Drive, Bethesda, MD 20892-5772 USA

**Keywords:** Biophysics, Materials science

## Abstract

Articular cartilage is a load-bearing tissue found in animal and human joints. It is a composite gel-like material in which a fibrous collagen network encapsulates large proteoglycan assemblies that imbibe fluid and “inflate” the network. Here we describe a composite hydrogel consisting of a cross-linked polyvinyl alcohol matrix filled with poly(acrylic acid) microparticles that mimics functional properties and biomechanical behavior of cartilage. The swelling and mechanical behaviors of this biomimetic model system are strikingly similar to that of human cartilage. The development of synthetic composite gel-based articular cartilage analog suggests new avenues to explore material properties, and their change in disease and degeneration, as well as novel strategies for developing composite tissue-engineered cartilage constructs for regenerative medicine applications.

## Introduction

This manuscript describes a predictive composite hydrogel model of cartilage. A novel aspect of the model is the incorporation of the pre-stress, which has been largely overlooked in previous tissue models. Pre-stress is present even in the absence of external loading, and it defines the load-bearing ability of cartilage. We demonstrate that the swelling and mechanical behavior of our biomimetic model system reproduces remarkably well the behavior of healthy and osteoarthritic human cartilage.

From a biological perspective, articular cartilage is a thin connective tissue (2 to 4 mm thick) that caps the ends of bones at joints. Cartilage extracellular matrix (ECM) consists mainly of proteoglycans (PGs), collagens, water, and ions. The most abundant macromolecule in cartilage ECM is Type II collagen, which is organized into fibrils that form a meshwork at larger length scales. The major PG in cartilage is the bottlebrush-shaped aggrecan that “inflates” the collagen matrix^1–5^.

**Figure 1:**
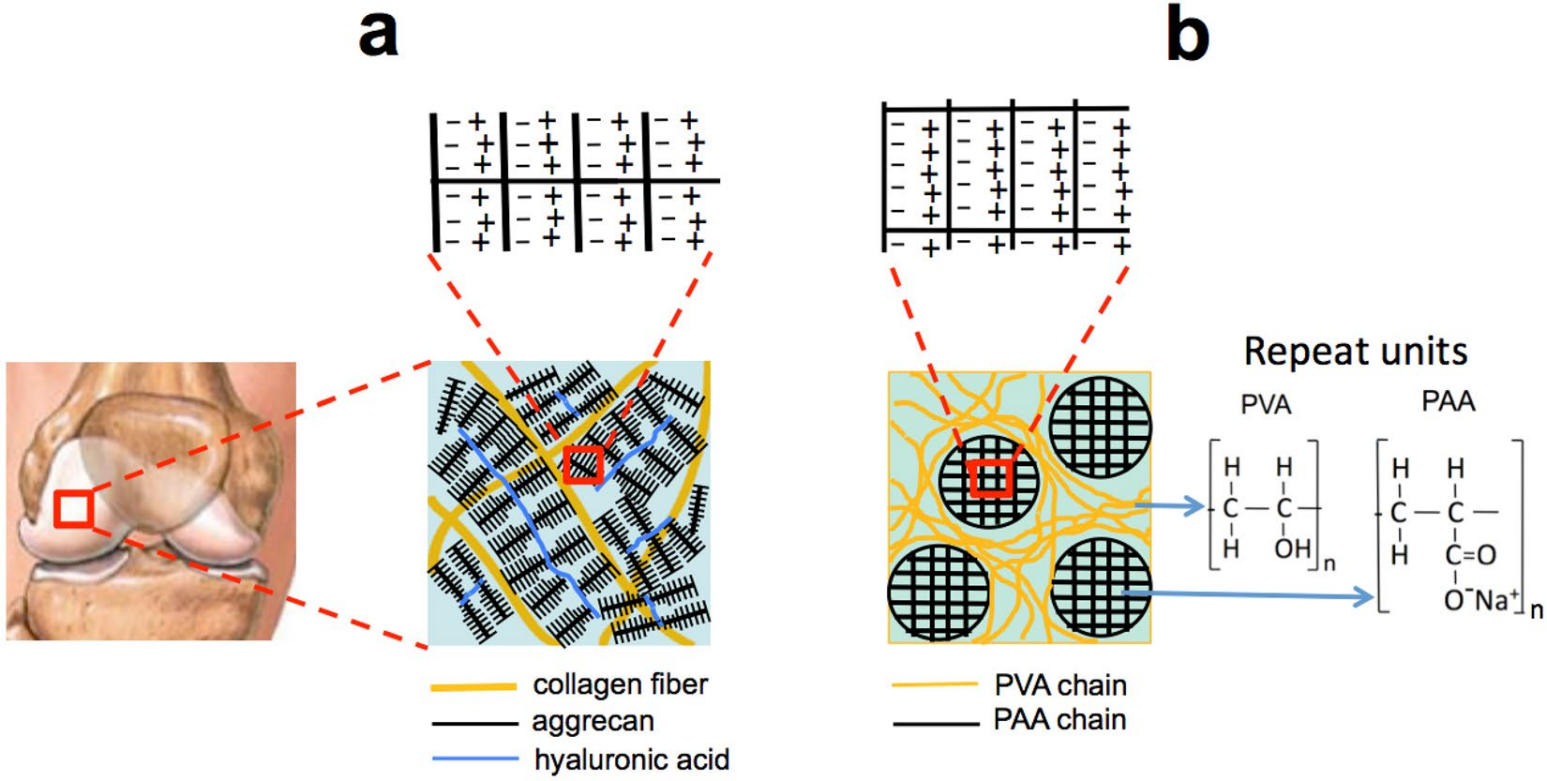
Schematic drawings of (**a**) cartilage ECM and (**b**) PVA/PAA composite gel. In both systems the chains are extended due to the electrostatic repulsive forces between the negatively charged groups. Also shown in (**b**) are repeat units of PVA and PAA chains; in PAA as the pH increases, hydrogen ions are replaced by Na^+^ ions.

Intracellularly synthesized aggrecan molecules are secreted into the ECM, where they aggregate to form a secondary bottlebrush with hyaluronic acid (HA) stabilized by a link protein^6–8^. The osmotic swelling pressure of the aggrecan–HA assemblies inflates the surrounding collagen matrix (Fig. 1a). At swelling equilibrium, the tissue is prestressed, conferring unique mechanical properties on cartilage. At equilibrium, although the total swelling pressure is zero, the collagen matrix is in tension. In diseases, such as osteoarthritis, the lubricating and load-bearing ability is reduced, however, the mechanisms that lead to loss of cartilage’s key biomechanical properties are still poorly understood.

From a polymer science perspective, cartilage is a composite load-bearing gel, consisting of a relatively stiff, fibrous collagen network that encapsulates large, negatively charged (anionic) proteoglycan (PG) polymer assemblies that exhibit a hierarchical bottlebrush organization. These PG polyelectrolytes draw water in osmotically, causing this phase to swell against the collagen network phase that confines them, increasing the overall stiffness of the cartilage tissue. Under a compressive loading, fluid is expelled from the tissue; after unloading it recovers its original shape and volume. The resistance of cartilage to such deformation and volume changes defines its load-bearing capacity. In degenerative joint diseases (e.g., osteoarthritis) cartilage structure is progressively damaged. Changes in the stiffness of the collagen matrix and/or loss of PGs strongly affect the biomechanical properties (e.g., load-bearing capacity) of the tissue^9,10^.

Although a qualitative picture of cartilage behavior being explained by the interplay between the swelling of the PGs and the restraint imposed by the collagen network was first proposed by Ogston^1,2^, a deeper polymer chemistry and physics-based understanding of the determinants of cartilage’s key functional properties, such as its load-bearing ability, has eluded adequate description.

Attempts have been made to describe the mechanical behavior of cartilage in terms of simplified physical models^11,12^. For example, the cartilage network has been likened to a network of strings that form a tethered fishnet which, when inflated by balloons trapped within it, can alter network tension and the stiffness of the composite material that is able support significant loads when these balloons are sufficiently inflated^13^.

It is also known that ‘prestress’ may considerably increase the load-bearing capacity of composite materials such as reinforced concrete, in which one component is in tension while the other is in compression. The tensegrity model also has both tensile and compressive elements^14,15^, and has been applied successfully to understanding the mechanics of cells.

Hydrogels with tunable swelling and mechanical properties have been widely used as models to mimic various biological tissues, including cartilage^4,16^. One of the basic challenges in constructing a biomimetic model of cartilage, however, is to incorporate prestress, which is present even in the absence of any external loading, and which plays a critical role in governing the tissue’s load-bearing ability.

This work focuses on developing a quantitative and qualitative understanding of load bearing in cartilage by viewing it as a composite gel. In particular, we propose and investigate a novel pre-stressed biomimetic composite gel model consisting of a poly(vinyl alcohol) (PVA) matrix, which encapsulates weakly cross-linked poly(acrylic acid) (PAA) microgel particles, in which the PVA network corresponds to the collagen matrix, while the PAA microparticles correspond to the PG polyelectrolye microgel phase (Fig. 1b). In equilibrium with pure water or in physiological salt solution, PAA absorbs water and inflates the PVA matrix. The retraction stress generated in the PVA matrix constrains the swelling of PAA in equilibrium, imbuing the composite medium with remarkably high stiffness despite that the two polymeric components are soft and ‘squishy’.

The present work exploits the differences between the swelling pressures of PVA and PAA gel networks to create a prestressed tissue model system that mimics the osmotic and mechanical behavior of cartilage. At the time of synthesis, both components are stress-free in the PVA/PAA composite gel. However, as the PAA swells, the PVA network is put under increasing tension. At the same time, the PAA component is under increasing compression. Although the unloaded composite material is in unloaded mechanical equilibrium, both components are deformed from their original equilibrium configurations. Here we investigate the swelling behavior of PVA/PAA composite gels, determine their prestress, and compare the swelling behavior of the composite biomimetic tissue with data reported for healthy and osteoarthritic cartilage we have previously obtained.

## Methods

### Preparation and characterization of composite PVA/PAA hydrogels

PVA is a neutral polymer, which is relatively insensitive to changes in the composition of the surrounding solution (e.g., salt concentration, pH). Chemically and/or physically cross-linked PVA gels are widely used in biomedical applications^17–19^. Many attempts have been made to create PVA gels with mechanical properties (stiffness, etc.) similar to those of cartilage^20–23^. In the present study, this goal was achieved by combining chemical cross-linking of PVA with partial crystallization induced by freeze thawing. PVA/PAA composite gels were prepared by dispersing PAA microgel particles in a PVA solution (1:1 ratio) prior to PVA cross-linking The size of the PAA particles was in the range of 5 to 10 μm Glutaraldehyde was used to cross-link the PVA molecules. The gels were equilibrated with 100 mM NaCl solution. The pH was varied by adding appropriate amounts of 0.1 M NaOH solution. Figure 1b shows the repeating monomeric units of PVA and PAA chains. (See details of gel preparation in Supplementary Materials).

The swelling pressure of the gels Π_sw_ was determined by an osmotic stress technique^24–26^. In this method gels enclosed in a dialysis bag are equilibrated with polymer solutions of known osmotic pressure^26^. At equilibrium, the concentrations of the gels and the polymer solutions are determined. This procedure yields the dependence of Π_sw_ on the polymer volume fraction φ (see more details in Supplementary Materials).

The present composite gel is a novel type of soft matter. It is different from the so-called double-network hydrogels, which consist of two polymer networks, a short-chain and a long-chain network. In a double network, the polymer components are strongly interpenetrated. However, in PVA/PAA composite gels the two networks are not interpenetrated. Furthermore, small angle neutron scattering (SANS) measurements indicated that the interaction between the matrix polymer and the encapsulated gel particles was negligible (see Supplementary Materials). We note that previous investigations showed the absence of significant interaction between the major polymeric components of cartilage ECM, collagen, and aggrecan^27^.

## Results

### Unique mechanical and osmotic properties of PVA/PAA composite gels

**Figure 2:**
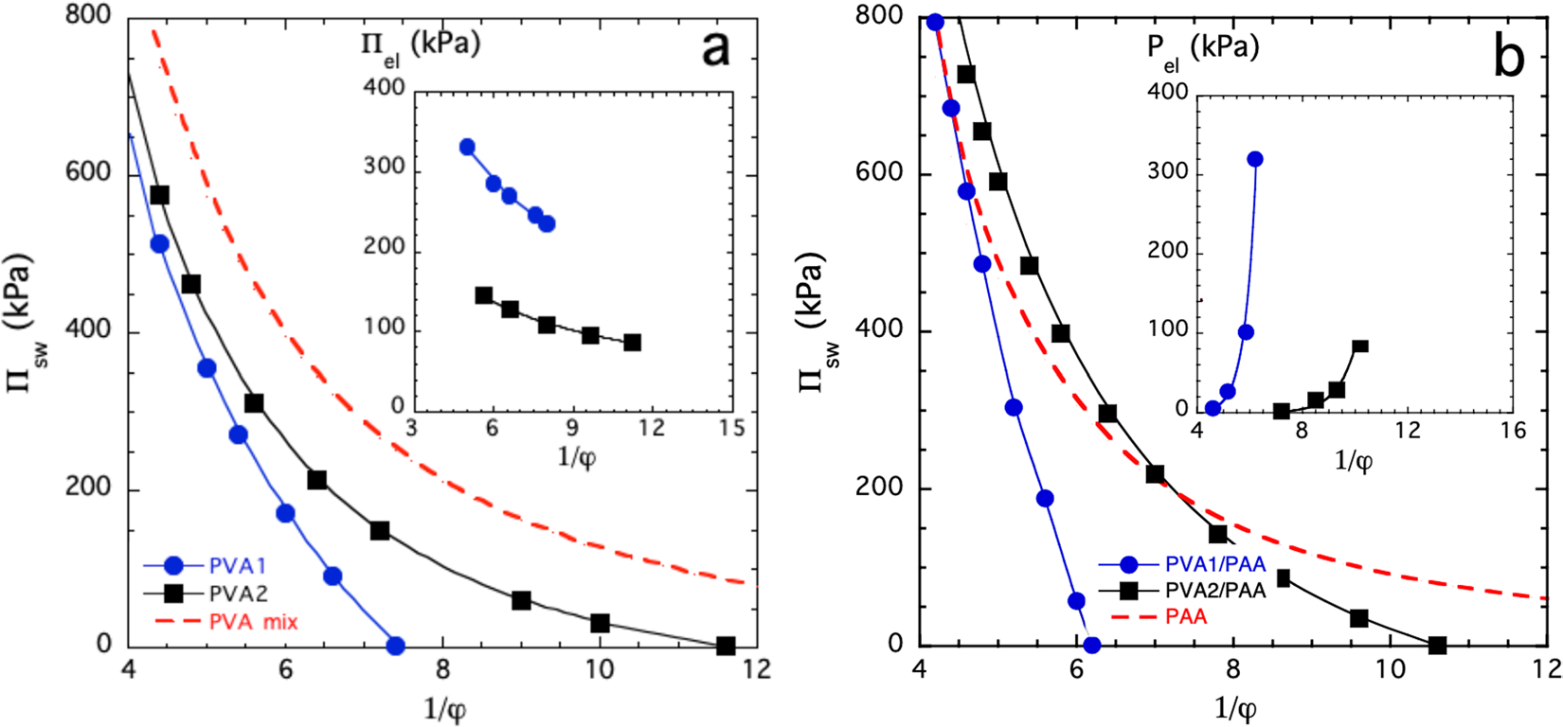
Π_sw_ versus 1/φ plots for (**a**) PVA and (**b**) PVA/PAA gels. The cross-link density of PVA1 is greater than that of PVA2. Continuous curves through the data points provide visual guides. The red dashed curves show Π_mix_ versus 1/φ for (**a**) the uncross-linked PVA and (**b**) the swelling pressure for the PAA particles enclosed in the PVA matrix. Insets: (**a**) Π_el_ versus 1/φ plots and (**b**) P_el_ versus 1/φ plots.

Figure 2 shows the variation of II_sw_ as a function of the swelling degree 1/φ (where φ is the volume fraction of the polymer in the swollen network) for PVA (Fig. 2a) and PVA/PAA (Fig. 2b) gels at pH = 7. The II_sw_ versus 1/φ curves for the PVA/PAA composite gels are steeper than for the pure PVA gels. The load-bearing ability of these gels is governed by their osmotic compression modulus K_os_ = φ∂II/∂φ, which is the measure of the resistance of the gel to a fractional volume change under load. The increase in the slope of the II_sw_ versus 1/φ plots implies that the load-bearing ability of the composite systems exceeds that of the PVA gels by a factor of approximately two.

The PVA/PAA gel appears to resemble an ordinary filled-polymer network. However, in filled polymers the filler particles (e.g., fumed silica) form links between the network chains, thus increasing the effective “cross-link density”. In the present composite gel the encapsulated gel particles are not chemically attached to the polymer matrix and, therefore, do not increase the number of contacts between neighboring polymer chains. Consequently, the reinforcing mechanism in the PVA/PAA gel should be completely different from that in regular filled-polymer networks.

The driving force of gel swelling is the osmotic pressure of the polymer within the gel, II_mix_, which causes the motion of solvent molecules from a solvent rich region to a region of lower solvent concentration. In the absence of cross-links, the polymer is completely dissolved (infinite swelling) due to thermodynamic interactions between the polymer and solvent molecules. However, cross-links prevent infinite swelling because the osmotic pressure of the polymer is counterbalanced by the elastic pressure, II_el_, generated by the cross-links. In regular polymer gels, the elastic pressure, II_el,_ decreases with an increasing degree of swelling reflecting the decrease of the cross-link density^28,29^. At equilibrium, the solvent flow stops, and the swollen polymer network coexists with the solvent. In other words, II_el_ counteracts II_mix_ and the swelling pressure II_sw_ is zero,

1 $${{\rm{II}}}_{{\rm{sw}}}={{\rm{II}}}_{{\rm{mix}}}-{{\rm{II}}}_{{\rm{el}}}=0$$

Equation 1 implies that in regular gels, II_mix_ is always greater than II_sw_, i.e., the II_sw_ versus 1/φ and the II_mix_ versus 1/φ curves never intersect each other. This behavior is illustrated in the inset of Fig. 2a for both a weakly and a densely cross-linked PVA gel. In both systems, II_el_ decreases monotonically with 1/φ.

Figure 2b shows that in composite gels the situation is entirely different. In these systems the encapsulated gel particles swell and inflate the surrounding matrix. At the concentration where the swelling pressure curves intersect each other, II_sw_ = II_PAA_ (where Π_PAA_ is the swelling pressure of the encapsulated PAA gel particles). At higher swelling degrees, the swollen gel particles produce a tensile stress, or prestress, P_el_, and the matrix becomes inflated. The prestress is defined as the difference between the swelling pressure of the enclosed polymer (Π_PAA_) and that of the composite gel (Π_sw_)

2 $${{\rm{P}}}_{{\rm{el}}}={{\rm{P}}}_{{\rm{PAA}}}-{{\rm{P}}}_{{\rm{sw}}}$$

The inset in Fig. 2b shows that in the PVA/PAA composite gels, P_el_ steeply increases with increasing swelling degree and reaches a maximum, P_el_^max^, when the composite gel is fully swollen, i.e., Π_sw_ = 0. At this concentration, the swelling pressure of the PAA is balanced by the prestress developed in the PVA matrix (Π_PAA_ = P_el_^max^). In the absence of external loading, the tensile stress in the PVA is equal to the compressive stress in the PAA and the net stress is equal to zero.

**Figure 3:**
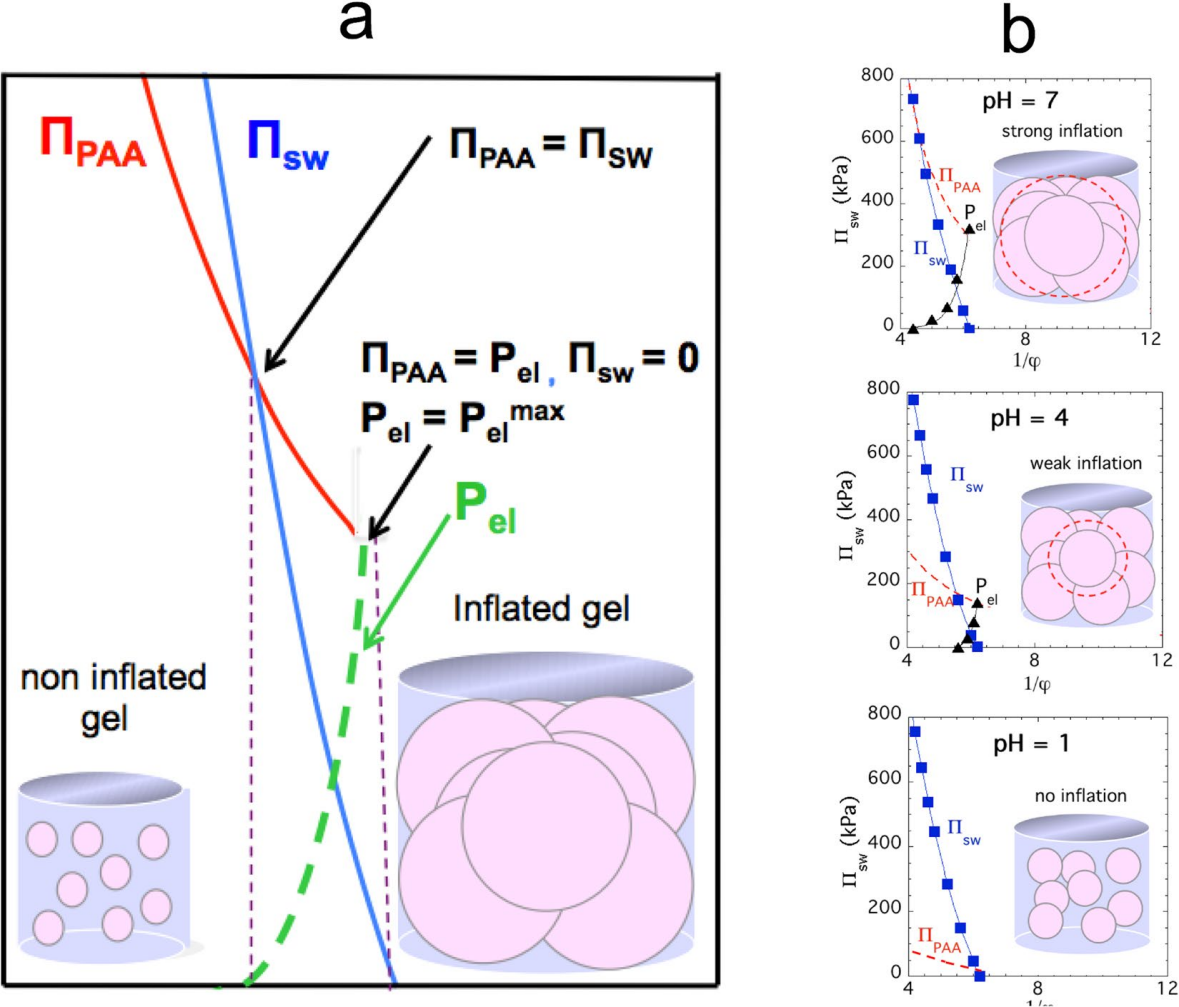
(**a**) Schematic representation of the osmotic components in a composite gel. At Π_PAA_ = Π_sw_, the prestress, P_el_ = 0. In the fully swollen gel, Π_sw_ = 0, and at this point Π_PAA_ = P_el_ = P_el_^max^. (**b**) II_sw_ versus 1/φ plots for PVA/PAA composite gels at different pH. The three insets show schematically the structure of the fully swollen composite gels (II_sw_ = 0). Matrix inflation increases with increasing pH. The dashed circles indicate the size of the freely swollen PAA particles. In the inflated gel, the PAA particles are smaller than the fully swollen particles due to the compressive stress imposed by the PVA matrix.

Although formally Eqs. 1 and 2 are similar, there is an important conceptual difference between P_el_ and II_el_. P_el_ increases with increasing swelling degree as shown in Fig. 2b (inset); this behavior is the opposite of the decrease of II_el_ observed in regular gels (inset in Fig. 2a). (The theory of rubber elasticity^28,29^ predicts that in an ideal gel II_el_ ∝ φ^1/3^). In other words, the high osmotic swelling pressure of the PAA offsets the reduction of II_el_, which implies that the inflated gel operates at an elevated stress level, i.e., the Π_sw_ = 0 condition is shifted upward along the *y*-axis. Because of the highly nonlinear character of the Π_sw_ versus 1/φ curves, both Π_sw_ and the actual swelling degree of the composite gel are reduced. The “strain-hardening” increases with increasing prestress and can be quantified by the osmotic compressional modulus of the gels. We note that the steep increase of P_el_ can be satisfactorily described by the phenomenological Fung hyperelasticity model^30,31^, which is widely used to analyze the load-deformation behavior of diverse biological materials. The situation in the composite gel is illustrated in Fig. 3a.

Figure 3b shows the effect of pH on the inflation of PVA/PAA composite gels at three values of pH (7, 4, and 1). The swelling degree of the PAA component increases with increasing pH and is highest at pH = 7. At this pH, the carboxyl groups are dissociated. At pH = 4, the swelling of the PAA particles is significantly less than at pH = 7, and at pH = 1, they hardly swell. At pH = 1, the PAA swelling pressure curve lies below the PVA/PAA curve over the entire concentration range, indicating that the carboxyl groups of the PAA are fully protonated and the swelling pressure vanishes. The absence of intersection implies that at pH = 1 no prestress is generated in the composite gel. The insets illustrate that the size of the PAA particles in the inflated gel is always smaller than their size when they are freely swollen. The inflated PVA matrix imposes a compressive stress preventing the PAA gel particles from reaching their freely swollen state.

### Comparison between the swelling pressure of PVA/PAA composite gels and cartilage samples

**Figure 4:**
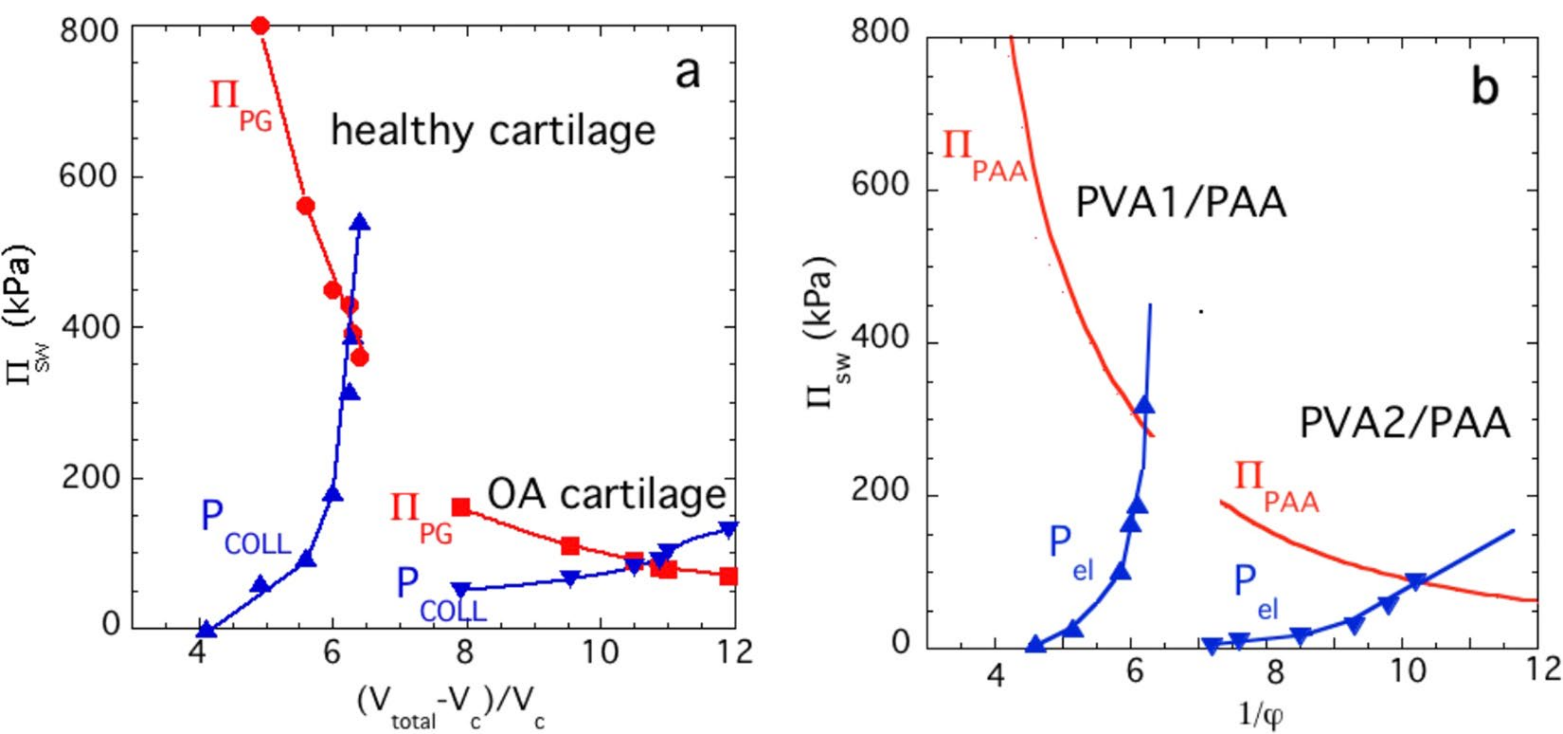
Comparison between (**a**) the swelling behavior of cartilage samples and (**b**) PVA/PAA composite gels. The *x*-axis represents the hydration of the cartilage, where V_total_ is the total tissue volume and V_C_ is the volume of the collagen. In (**a**) II_PG_ is the osmotic pressure of the PG component and P_COLL_ is the tensile stress of the collagen network.

The ultimate test of a biomimetic model is to compare its behavior with its tissue counterpart. For this comparison, we used high-quality osmotic swelling pressure data we previously reported for cartilage (Fig. 4a)^32^ where we investigated the effect of hydration on the tensile stress for normal (healthy) human cartilage samples and for cartilage from an osteoarthritic (OA) joint. Cartilage was taken from femoral heads at operations for femoral neck fractures and at post mortem (normal: 55 years) and (OA: 68 years)^32^. In healthy cartilage, the P_el_ versus hydration curves exhibited a steep increase with increasing hydration. By contrast, the swelling pressure curve for the OA specimen was significantly less steep and displaced to higher hydrations. These findings indicate that in OA cartilage the collagen network is weaker. Owing to its high water content, the OA cartilage cannot develop a high PG concentration and, therefore, tissue load-bearing capacity is significantly reduced.

Figure 4b shows that the osmotic response of the PVA/PAA composite hydrogels is similar to that of the cartilage tissue. The biomimetic model reproduces both the shape of the cartilage swelling curves and the actual values of the swelling pressure reported for normal and OA tissues.

### Biological relevance and perspectives

Load-bearing tissue is ubiquitous in nature. In ECM, the composition and structural organization of its components are critically important because their biochemical and mechanical properties and the complexity of the tissue architecture control the tissue’s overall mechanical properties, such as the load-bearing ability.

The main objectives of the field of tissue sciences are to develop quantitative relationships between tissue structure, composition, and its functional properties, identifying and quantifying normal behavior and understanding determinants of pathological deviations from normal behavior. Systematic studies on biomimetic hydrogels could provide vital insights to help understand how factors (e.g., matrix stiffness, charge density) affect the macroscopic mechanical and swelling properties of tissues. This understanding cannot be obtained from measurements made on biological samples because their composition and physical properties cannot be independently and systematically varied as they can be in biomimetic model systems.

Prestress is a key mechanical property of load-bearing biological tissues. Although, its importance has been recognized, its consequences have not yet been fully explored. For example, tissue prestress is clearly a critical factor when designing and engineering a competent cartilage implant or when considering tissue regeneration strategies. Cartilage load-bearing ability is determined by the relative amounts of collagen and PGs and by their structural integrity. The ability of collagen to resist tension confers the shear stiffness of the tissue. PG assemblies inflate the collagen matrix and generate prestress in the collagen network ensuring that the tissue operates in the inflated state (i.e., with increased matrix stiffness) and exhibits enhanced load-bearing properties. High collagen content increases matrix stiffness and, therefore, it is a basic requirement for effective load-bearing^33,34^.

There are various biochemical methods to control the collagen-to-proteoglycan ratio. For example, catabolic enzymes (e.g., chondroitinase, hyaluronidase) deplete PG concentration and increase the tensile properties of cartilage^35^. Suppression of PG synthesis may establish an environment favorable for engineering a deflated matrix. However, reducing the PG concentration also reduces the prestress in the matrix and ultimately decreases the stiffness of the tissue. The present biomimetic model makes it possible to systematically investigate the effect of different factors and trade-offs for developing successful tissue engineering strategies that optimize tissue properties^36^.

Another potential application of this modeling framework relates to the treatment of degenerative joint diseases (e.g., OA) by the injection of hyaluronic acid gel particles to reduce osteoarthritis pain^37,38^. In previous studies, neither the effect of the cross-link density nor the size of the HA particles has been optimized. Our biomimetic gel model predicts that load-bearing properties can be maximized by tailoring the cross-link density of the HA gel. Furthermore, the particle size should also be carefully controlled to enable the particles to penetrate the collagen matrix and inflate it.

## Conclusions

The present work describes a biomimetic hydrogel model of cartilage consisting of a stiff PVA matrix in which PAA microgel particles are embedded. This composite system exhibits entirely different mechanical properties than regular gels. The most remarkable difference is the increase of the elastic pressure with increasing swelling degree. The PAA particles inflate the PVA matrix as the composite gel swells. At equilibrium, the swelling pressure of the PAA is compensated by the tensile stress developed in the PVA and the net “tissue” stress is zero. At pH = 7, the PAA particles absorb more liquid than the PVA and inflate the matrix. When the pH is reduced, the swelling pressure of the PAA is diminished due to the increased protonation of the carboxyl groups on the PAA chains, while the swelling of the PVA matrix is practically unchanged because the PVA does not contain ionizable groups and thus will not swell or shrink.

Analysis of the swelling pressure curves of the PVA/PAA composite gels reveals strong similarities with the behavior of cartilage. Our biomimetic hydrogels reproduce not only the shape of the cartilage swelling pressure curves but also the numerical values reported for healthy and osteoarthritic cartilage samples.

The present results illustrate that systematic studies on well-defined biomimetic model systems may provide important insight into the unique behavior of tissues that cannot be obtained from measurements made on biological samples.

## Supplementary information

Supplementary Information.
