# Supplementary material for: Composite Hydrogel Model of Cartilage Predicts Its Load-Bearing Ability
Source: Sci Rep. 2020 May 15;10:8103. doi: 10.1038/s41598-020-64917-1 (PMC7228937; doi:10.1038/s41598-020-64917-1)
Supplement: Supplementary file 1 — Supplementary Information. [file 41598_2020_64917_MOESM1_ESM.docx]

Supplementary Materials for

**Composite Hydrogel Model of Cartilage Predicts Its Load-Bearing Ability**

**Authors:** Ferenc Horkay, Peter J. Basser

Materials and Methods

*Gel preparation*

Poly(vinyl-alcohol) (PVA) gels were prepared by cross-linking with glutaraldehyde (GDA) at pH = 1.0 in aqueous solutions. The pH was adjusted by HCl solution. For the experiments, a fully hydrolyzed and fractionated PVA sample was used (M_w,PVA_ = 110 kDa). Cross-links were introduced at 4% (w/w) polymer concentration; the molar ratio of monomer units to the molecules of cross-linker was 200. After gel formation, the samples were equilibrated with 100 mM NaCl solution to remove HCl and uncross-linked polymer. Then the gels were dried, reswollen in 100 mM NaCl solution, and exposed to two (PVA2) and eight (PVA1) cycles of freezing for 12 to 14 hours at –20°C and thawing for 10 hours at 25°C.

Poly(acrylic acid) gels were synthesized by free-radical copolymerization of neutralized acrylic acid and *N*,*N*′-methylenebis(acrylamide) in aqueous solution. Special molds were used to make cylindrical (1 cm height, 1 cm diameter) gels. The monomer concentration was 30% (w/w). After the components were mixed, dissolved oxygen that would inhibit the polymerization reaction was eliminated by bubbling nitrogen through the solution. The polymerization reaction was initiated by ammonium persulfate (0.5 g/L). Gelation was achieved at 80 °C. Gels were kept at 80 °C for 2 h and then were allowed to set at room temperature for 20 h to ensure that the reaction was complete. Gel cylinders were removed from the mold, neutralized fully, and placed in deionized water to remove any unreacted materials and other components (sol fraction) not attached to the network. Water was replaced every day for 2 weeks. The polymer gel was dried at 95 °C, and the dry polymer was ground into powder having an average particle size < 10 microns measured by optical microscopy.

PVA/PAA composite gels were prepared by cross-linking PVA in solution containing PAA particles. First, HCl was added to the solution (pH ≈ 1) to suppress the swelling of PAA. Then, the suspension was cross-linked by GDA. The molar ratio of the monomer units to the cross-linker was the same as in the case of the pure PVA gels. The composite gels were treated to the same freeze–thaw process as the PVA gels.

*Osmotic stress measurements*

Deswelling of the gels was achieved by enclosing them in a semipermeable membrane (dialysis bag, seamless cellulose tubing; cut off molecular weight: 12 kDa, Sigma Chemical Co., St. Louis, MO). Solutions of known concentrations of an osmotic deswelling agent [poly(vinyl pyrrolidone), PVP, Mn: 29 kDa] were equilibrated with the gel for 5-10 days. The semipermeable membrane prevented penetration of PVP into the gel. At equilibrium, the swelling pressure II_sw_ of the gel inside the dialysis bag is equal to the osmotic pressure of the PVP solution outside [18]. Periodically the mass of the gel m_g_ was measured, and used to calculate the total polymer weight fraction C = m_d_/m_g_ (where m_d_ is the mass of the dry gel). The volume fractions were calculated from the known densities of the polymers (PVA: 1.27 g/cm^3^, PAA: 1.32 g/cm^3^) and the solvent (water: 0.997 g/cm^3^). Equilibrium was achieved when no further changes in either gel swelling degree or solution composition were detectable. The gel samples were dried at 95 ^o^C. Reversibility was checked by transferring the gels into PVP solutions at different osmotic pressure values.

Swelling pressure measurements were made on at least 3 nominally identical gel samples at 25 ± 0.1 ^o^C. Repeated measurements showed a mean change in the osmotic swelling pressure less than 5%.

*Mechanical measurements*

The shear moduli of the PVA and PAA gels were determined from uniaxial compression measurements made on isometric gel cylinders (height = diameter) by a TA.XT2I HR texture analyzer (Stable Micro Systems, U.K.). This apparatus measures the deformation as a function of the applied force. Cylindrical gel specimens were uniaxially compressed (at constant volume) between two parallel flat glass plates. Typical sample sizes were 1-2 cm in height and 1-2 cm in diameter. Measurements were made in the range of deformation ratios 0.7 < Λ < 1 (Λ = *L*/*L*_o_, *L* and *L*_o_ being the lengths of the deformed and undeformed gel specimen, respectively). The elastic (shear) modulus, G_,_ was calculated from the nominal stress, σ (force per unit undeformed cross section), using the equation

σ = G (Λ - Λ^-2^) (S1)

Typical duration of a stress-strain measurement was between 5 and 10 minutes. The mechanical measurements were carried out at 25 ± 0.1 °C. No volume changes or barrel distortion were detected. The reproducibility of the whole procedure, including gel preparation, and modulus measurements was found to be within 4-5%.

Figure S1 shows typical -σ/(Λ - Λ^-2^) vs Λ^-1^ data for PVA gels at different degrees of swelling. In these gels G decreases as the swelling degree increases.


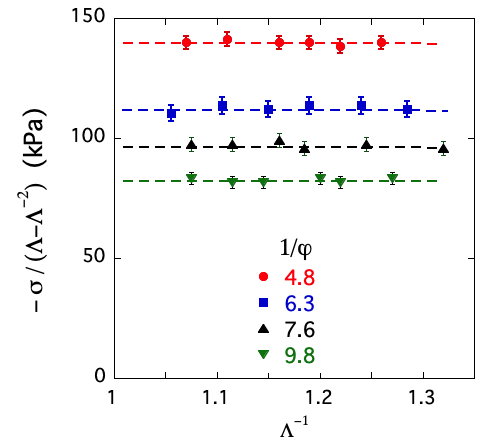


Figure S1. Typical -σ/(Λ - Λ^-2^) vs Λ^-1^ plots for a PVA gel at different degrees of swelling 1/ϕ.

In Figure S2 is plotted Π_sw_ (determined from osmotic swelling pressure measurements) as a function of G. The agreement found between the values of II_el_ obtained from osmotic swelling pressure measurement, and G determined from uniaxial compression measurements indicates that the two methods are equivalent.


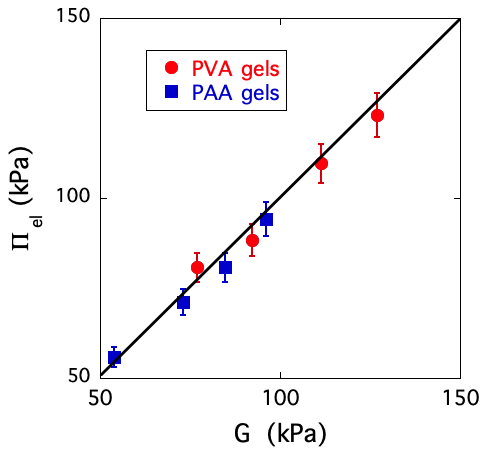


Figure S2. Dependence of II_el_ obtained from osmotic swelling pressure measurement on the shear modulus determined from uniaxial compression measurements for PVA (red symbols) and PAA gels (blue symbols).

*Small angle neutron scattering (SANS) measurements*

SANS measurements were made on the NG3 instrument at NIST, Gaithersburg MD. The incident wavelength was 8 Å. The sample-detector distances were 1.2, 4, and 13.1 m corresponding to a wave vector range 0.005 Å^-1^ < *q* < 0.1 Å^-1^. The ambient temperature during the experiments was 25 ± 0.1^o^C. Gel samples were prepared in D_2_O solutions in 2-mm thick sample cells. After azimuthal averaging, corrections for incoherent background, detector response, and cell window scattering were applied.

*SANS on PVA and PAA gels and on PVA/PAA composite gel*

Eq. 2 is valid only if there is no interaction between the matrix polymer (PVA) and the enclosed gel particles (PAA). This assumption was validated by making SANS measurements that probe the structure and interactions over a broad range of length scales extending from a few Å to approximately 500 Å. Figure S3 shows the SANS profiles of the two individual polymers (PVA1 and PAA) together with that of a PVA1/PAA composite system. The dashed curve through the data points of the composite gel is calculated by assuming additivity of the contributions of the PVA1 and PAA components. The agreement between the experimental and calculated data indicates that in the PVA1/PAA system the interaction between the PVA1 matrix and the enclosed PAA gel particles is negligible.

Figure S3. SANS profiles of PVA1, PAA and PVA1/PAA composite gels. The intensity of the PVA1/PAA curve is multiplied by 10 for clarity. The dashed curve through the PVA1/PAA data points is the sum of the SANS curves of the individual components of the same overall polymer concentration. Total polymer concentration: 8% (w/w).
